# Adverse Effects of COVID-19 Vaccination: Machine Learning and Statistical Approach to Identify and Classify Incidences of Morbidity and Postvaccination Reactogenicity

**DOI:** 10.3390/healthcare11010031

**Published:** 2022-12-22

**Authors:** Md. Martuza Ahamad, Sakifa Aktar, Md. Jamal Uddin, Md. Rashed-Al-Mahfuz, A. K. M. Azad, Shahadat Uddin, Salem A. Alyami, Iqbal H. Sarker, Asaduzzaman Khan, Pietro Liò, Julian M. W. Quinn, Mohammad Ali Moni

**Affiliations:** 1 Department of Computer Science and Engineering, Bangabandhu Sheikh Mujibur Rahman Science and Technology University, Gopalganj 8100, Bangladesh; 2 Department of Computer Science and Engineering, University of Rajshahi, Rajshahi 6205, Bangladesh; 3 Department of Mathematics and Statistics, Faculty of Science, Imam Mohammad Ibn Saud Islamic University (IMSIU), Riyadh 13318, Saudi Arabia; 4 Complex Systems Research Group, Faculty of Engineering, The University of Sydney, Darlington, NSW 2008, Australia; 5 Department of Computer Science and Engineering, Chittagong University of Engineering & Technology, Chittagong 4349, Bangladesh; 6 School of Health and Rehabilitation Sciences, Faculty of Health and Behavioural Sciences, The University of Queensland, St Lucia, QLD 4072, Australia; 7 Computer Laboratory, The University of Cambridge, 15 JJ Thomson Avenue, Cambridge CB3 0FD, UK; 8 Healthy Ageing, The Garvan Institute of Medical Research, Darlinghurst, NSW 2010, Australia

**Keywords:** COVID-19, vaccination, adverse reactions, comorbidities, symptoms, machine learning, statistical analysis

## Abstract

Good vaccine safety and reliability are essential for successfully countering infectious disease spread. A small but significant number of adverse reactions to COVID-19 vaccines have been reported. Here, we aim to identify possible common factors in such adverse reactions to enable strategies that reduce the incidence of such reactions by using patient data to classify and characterise those at risk. We examined patient medical histories and data documenting postvaccination effects and outcomes. The data analyses were conducted using a range of statistical approaches followed by a series of machine learning classification algorithms. In most cases, a group of similar features was significantly associated with poor patient reactions. These included patient prior illnesses, admission to hospitals and SARS-CoV-2 reinfection. The analyses indicated that patient age, gender, taking other medications, type-2 diabetes, hypertension, allergic history and heart disease are the most significant pre-existing factors associated with the risk of poor outcome. In addition, long duration of hospital treatments, dyspnoea, various kinds of pain, headache, cough, asthenia, and physical disability were the most significant clinical predictors. The machine learning classifiers that are trained with medical history were also able to predict patients with complication-free vaccination and have an accuracy score above 90%. Our study identifies profiles of individuals that may need extra monitoring and care (e.g., vaccination at a location with access to comprehensive clinical support) to reduce negative outcomes through classification approaches.

## 1. Introduction

The Severe Acute Respiratory Syndrome Coronavirus 2 (SARS-CoV-2) variants give rise to COVID-19, the pandemic disease that has caused a massive public health emergency worldwide since the first reports in December 2019 in Wuhan, China [1,2]. SARS-CoV-2 virus is genetically related to a number of coronaviruses found in bat species, and its genetic sequence matches 79% and 50% with the coronaviruses responsible for severe acute respiratory syndrome (SARS) and the Middle East respiratory syndrome (MERS) [3], respectively. As of December 2022, approximately 579 million people have been infected, leading to 6.6 million fatalities [4]. Some forecasts of the number of COVID-19 cases using Bayesian regressions may help governments to take actions to avoid the disease’s spread [5,6,7]. Vaccines against SARS-CoV-2 were developed rapidly and are now in wide use. These are the first fully validated vaccines against coronavirus infections in humans [8], although vaccines for coronaviruses responsible for some nonhuman diseases have previously been developed [9]. Attempts had been made to create vaccines for SARS and MERS, but these so far have been tested only in nonhuman species [10,11].

Approximately 356 candidate vaccines for SARS-CoV-2 have been developed, of which (by December 2022) 39 of these are undergoing phase I trials, 32 are in combined phase I/II trials, 18 in phase II trials, 40 in phase III trials, and 9 in licensed use and phase IV observation trials; in total 138 have undergone or are undergoing clinical testing [12]. Many SARS-CoV-2 vaccines that have completed phase III trials have shown effectiveness in preventing death or serious disease that is above 90% for the original SARS-CoV-2 variant [12]. Currently approved vaccines include two Ribonucleic acids (RNA) vaccines (BNT162b2 from Pfizer–BioNTech and the mRNA-1273 vaccine from Moderna), four conventional inactivated virus vaccines (BBIBP-CorV from Sinopharm, BBV152 from Bharat Biotech, CoronaVac from Sinovac, and WIBP from Sinopharm), three adenoviral vector vaccines (Sputnik V from the Gamaleya Research Institute, the AZD1222 from Oxford–AstraZeneca, and Ad5-nCoV from CanSino Biologics), as well as a virus peptide fragment-based vaccine EpiVacCorona from the Vector Institute [12].

Numerous countries have run vaccination programs since early 2021 that prioritize individuals with the highest probability of severe complications from COVID-19, such as people of advanced age, and those at high risk of virus exposure and transmission, such as frontline medical staff [13]. As of March 2022, over 10.5 billion doses of the COVID-19 vaccine have been administered worldwide [12]. However, while vaccines protect from serious illnesses, they commonly demonstrate a small number of significant adverse reactions and side effects in mass administration programs. For COVID-19 vaccines, this has been noted particularly in individuals with significant pre-existing comorbidities, such as *diabetes and high blood pressure* and *allergic conditions*. This has led to significant vaccine hesitancy in the early vaccination period [14].

One issue arising particularly in rapid vaccine deployment is the difficulty in appraising the likelihood of adverse reactions to the vaccines in large populations [15,16]. Rarely occurring risk factors are, by the nature and size of the trials and limitations of time, unlikely to be seen in randomized clinical trials. Clinical and demographic information at the individual level can also affect vaccine response. While vaccine adverse reactions are a separate issue from vaccine effectiveness they may share some common factors, such as the strength of the immune response to the vaccine. Adverse reactions reported to date are rare, but some rare but serious cases of anaphylaxis, which can develop within minutes to hours after vaccination have been reported [17]; in addition, significant numbers of thrombolytic thrombocytopenia have been reported. Physicians and researchers at the US Centers for Disease Control and Prevention (CDC) assessed adverse reactions after vaccination to identify these reports as *anaphylaxis* or *not anaphylaxis* [18]. Thus, for example, in the USA, 1,893,360 people received their first dose of a COVID-19 vaccine in the week of 14 to 23 December 2020 [19], among which 21 cases were reported to have anaphylaxis responses by CDC; of these 21, 4 were hospitalized and 17 were treated in an emergency department [20]. Machine Learning (ML) based analyses have been performed to identify potential factors for several disease conditions [21], but this has not yet been performed for the COVID-19 vaccine reaction data.

There have been a small number of fatalities post COVID-19 vaccination [15,16,22], although the degree to which they are linked to the vaccination itself is unclear and still under active investigation [23,24]. Nevertheless, there is a chance of side effects with any medication administered to a very large population, necessitating close surveillance to detect any evidence of direct or indirect effects. While the number of adverse reaction cases to COVID-19 vaccination is extremely small in number relative to the number vaccinated, they cannot be overlooked as they give important information to predict and ameliorate adverse reactions and poor outcomes. Statistical and ML analysis [25] can play a role in characterizing those factors. We have, therefore, analyzed data from patients to clarify the common causes of such reactions. We employed statistical analysis and trained ML models to identify individuals most at risk of vaccine complications. If the causes of adverse effects of a vaccine are identified and eliminated and patients identified as at risk of complications are vaccinated in a safe medical environment, it would prevent the development of serious conditions and enable rapid treatment for anaphylaxis or other conditions, making COVID-19 vaccination safer.

The main objectives of this study are as below:

- To identify the most significant features of a patient’s past medical history that are associated with adverse effects of COVID-19 vaccination;
- To find the most significant patient symptoms that can predict the patient’s need for hospitalization for treatments after COVID-19 vaccination;
- In cases of death recorded after COVID-19 vaccination, to find the contributing causes of death;
- To identify and classify by the machine learning methods those patients that are at high medical risk of severe adverse reactions after COVID-19 vaccination and may need extra precautions.

## 2. Methods

In this study, we considered COVID-19 vaccinated patient data, including the past medical history, and their postvaccination effects and outcomes, and conducted data analyses by applying statistical methods and machine learning models. We also quantify the feature importance values to rank the features after model training.

### 2.1. Data Collection

In this study, initially, we have used a raw dataset of vaccinated USA patients that contains various kinds of vaccine-related information. The dataset was collected from the Vaccine Adverse Event Reporting System (VAERS) of observed individuals from December 2020 to 16 February 2022, who had reported adverse reactions after vaccination [26], from which a subset of 102,577 individuals was randomly chosen for further analysis. It contains information including COVID-19 vaccination status and the reactions to different sicknesses after vaccination. However, any non-COVID-19 information was omitted from our current study. In this dataset, for the most frequently used mRNA COVID-19 vaccines, the total number of collected reports was 72,147. VAERS collected the patient information on *age, gender, comorbidity history, allergic history, and birth defect information after vaccination, vaccination date, date of reaction onset, hospitalization information after onset, death event, recovery status, and laboratory test information after onset*. Additionally, the dataset also contains information about vaccine dose, days to onset, medical history, allergic history, type-2 diabetes status, and a list of medical history and reactions shown in the Table 1 and Table 2. All this information was included in the dataset obtained from VAERS and also used in this study.

This dataset has several limitations, but it is a warning system for further inquiry; therefore it could be helpful for analyzing these effects for monitoring purposes. Any afflicted individuals can report to VAERS using either an online platform or a paper document. Experts in vaccination safety analyze all reports of significant adverse events submitted to VAERS after receiving the report. Includes permanent impairment, hospitalization or an extended hospital stay, life-threatening disease, birth defects, and death. Due to the fact that the events are self-reported, some reports may contain incomplete, coincidental, erroneous, or unverifiable information. We have employed three sorts of indications for vaccinated candidates, including hospitalization, SARS-CoV-2 positivity, and death, so these instances are critical and will hopefully be monitored by VAERS specialists.

### 2.2. Data Processing

Before applying statistical methods and machine learning models, we preprocessed the dataset, including the use of feature extraction and feature engineering. After discussing this dataset with expert clinicians, we first constructed a designated list of features, e.g., symptom names and comorbidities. Then, we generated a keyword list with the help of those clinicians and applied string matching algorithms to prepare the dataset with features that included symptoms, aftereffects, and comorbidities.

Applying string matching and keyword selection techniques, we have extracted the patient medical history, such as pre-existing noncommunicable and communicable diseases, which included *hypertension, diabetes, chronic obstructive pulmonary disease (COPD), kidney disease, depression, and asthma* (detail is shown in Table 1). We have also included the reported adverse reactions, including the types of symptoms and signs such as *cough, high temperature, fatigue, fever, pyrexia, nausea, facial paralysis, and vomiting* (detail shown in Table 2). We thus obtained a processed dataset with 86 attributes and 72,147 entities.

In the data processing step, especially in feature extraction, we have considered some factors. Initially, we extracted and transformed values from the raw textual dataset [26], i.e., in the “gender” field, there were three types of values, i.e., `M’ as male, `F’ as female, and `U’ as unknown gender. In the `died’ and `disabled’ fields, we have considered `Y’ as yes and the remainder as `no’; in the `prior vaccine’ fields, mentioned the vaccine name as `yes’ and the rest are `no’. In the `allergic history’ field, we have considered mentioned allergic effects as a positive case of allergic history and the null values, values with `no’, `none’, `NA’, `no known allergic effects, and also more negatively mentioned text as a negative case. However, in the `History’ column in the raw dataset, coexisting conditions of patients were in written form. We extracted all of the patient’s medical histories separately. In this case, we have selected the keywords for each of the features and then matched them with the text and found the appropriate medical history, which we have considered as the most frequent top 27 individual medical histories. In the raw dataset, [26], there was a separate file that contains the patients’ adverse reactions as symptoms, including a key of `VAERS ID,’ where we have separated each of the 56 most frequent reactions, those are 56.22% developed within 24 h. There were three different files that were included in the dataset: the first one was for patients’ demographic and medical history, the second one was for patients’ reactions, and the final one was for vaccine information. We have merged the dataset according to the primary key `VAERS ID’. Finally, we have eliminated all of the non-COVID-19 vaccinated patients’ data.

**Figure 1:**
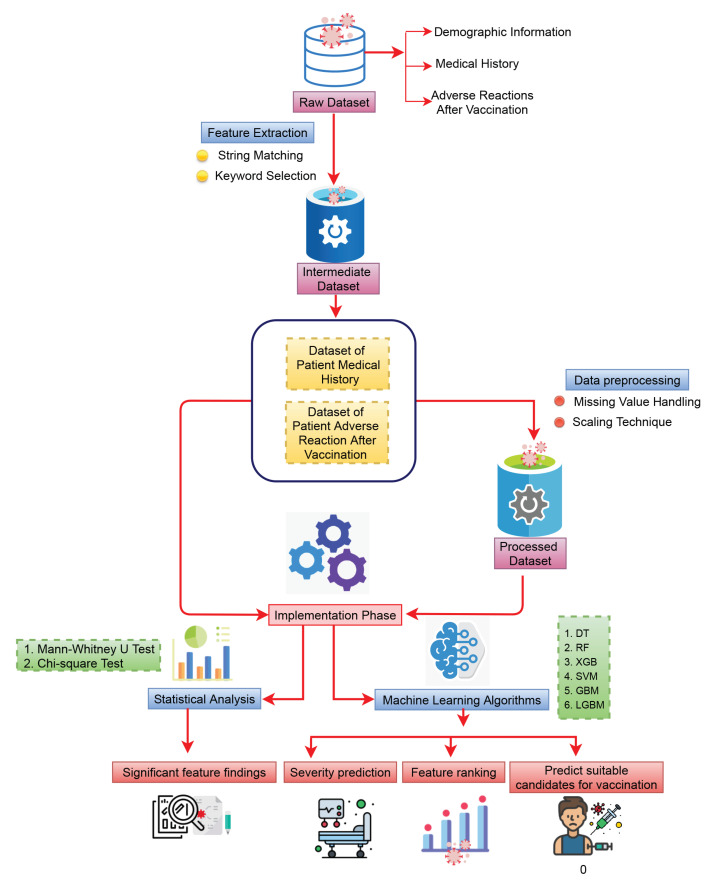
The schematic diagram of the overall workflow including data processing, data division, analysis using statistical and machine learning methods, and, at the end, performance evaluation with finding significant features.

We have partitioned our dataset into two different parts. The first part contained the patient medical history, and the second part consisted of the patient adverse reactions after vaccination (detail of the workflow is shown in Figure 1). After vaccination, some patients died shortly after developing some symptoms, some were re-infected with COVID-19, and some had shown sufficiently severe adverse reactions to require admission to hospital facilities for treatment. For this reason, we consider the three different types of target variables for patient comorbidities and reaction analysis after vaccination. The first one is “death status” (2348 were dead and others are alive), the second one is “SARS-CoV-2 test status” (13,546 were infected with COVID-19 and others are not), and the third one is patient “hospital admission status” (together 11,266 individuals were with severe reactions (all of them were hospitalized) and the others (who were not hospitalised))—all of which were observed after vaccination.

**Figure 2:**
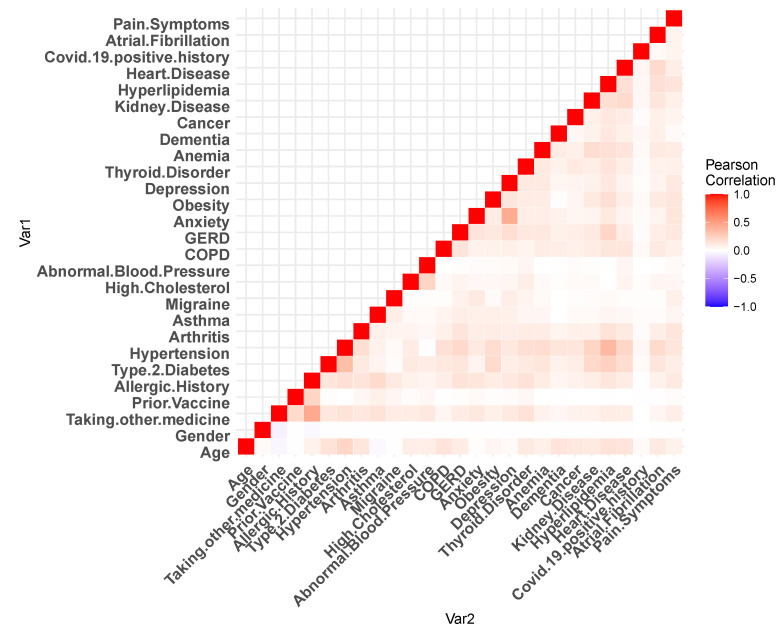
Pearson’s correlation heat-map for the dataset medical history.

**Figure 3:**
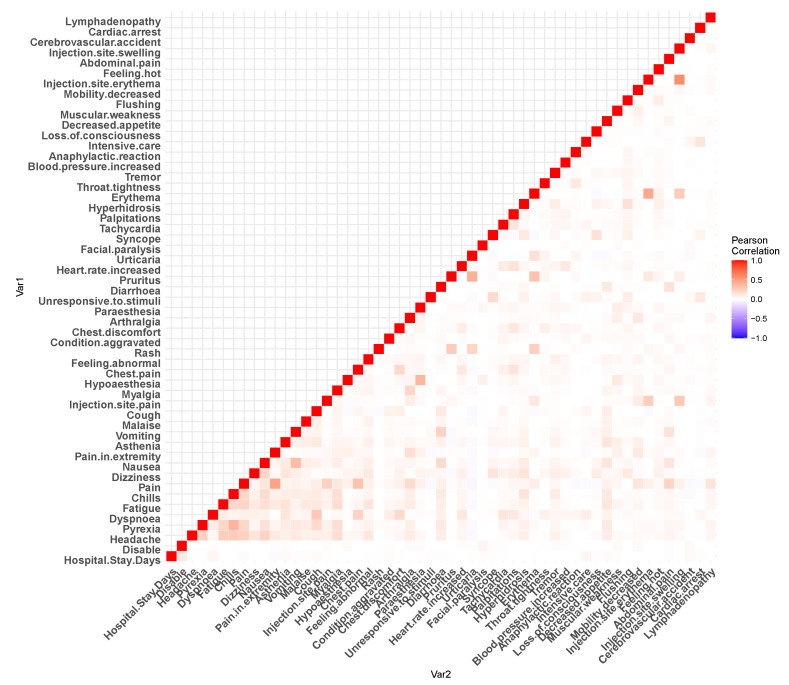
Pearson’s correlation heat-map for the dataset adverse reactions.

Figure 2 and Figure 3 show the Pearson’s correlation heat-maps for the patient medical history and reactions, respectively. Furthermore, for the machine learning algorithms, we have performed some additional steps to process the data. For the data field, namely *the age*, approximately 2.27% of data were missing, which was imputed with the mean value. Before each of the *train–test split* of the dataset, we have standardized our dataset with zero mean and unit standard deviation [27].

Among all of the 72,147 COVID-19 vaccinated individual patients, first, we have considered 2348 patients who died and 69,799 (72,147 − 2348) alive and completed the experiments. In the second phase, we repeated the experiment with a new set of data that includes 13,546 reinfected COVID-19 cases and controls (58,601 nonreinfected candidates), in this instance, we examined the control group whose COVID-19-positive status was negative. Altogether, 11,266 individuals were with severe reactions (all of them were hospitalized) and the others (who were not hospitalised) constitute the final sample for this study. In all of the experiments, we considered those attributes as independent variables and performed statistical and machine learning analyses.

### 2.3. Statistical and Machine Learning Approaches

We have used statistical and machine learning approaches to find the significant features. Machine learning models are also capable of distinguishing between the various group of patients. For the categorical variables, we used the chi-squared test to find the corresponding *p* values and consider p<0.05 as a significant as well as an associative parameter. Since age is absolute discrete data, we used the Mann–Whitney U test over two different populations.

We also performed descriptive statistical analysis to calculate the percentage and mean values of the features. In machine learning analysis, there are a range of models, i.e., decision tree (DT) and random forest (RF) (tree-based algorithms), support vector machine (SVM) are kernel-based and three boosting algorithms—gradient boosting machine (GBM), extreme gradient boosting machine (XGB) and light gradient boosting machine (LGBM) [28]. We selected those supervised machine learning algorithms for classification because of their excellent performance and quick execution [29]. For this purpose, classifiers that are based on max-voting, averaging, and weighted-averaging have been used as a basic ensemble learning approach. Along with that, the advanced ensemble learning approach also functions as stacking, bagging, and boosting. Those techniques are highly efficient and easy to debug [30].

In the model training phase, the machine learning algorithms had some parameters to classify and extract significant features. In the decision tree algorithm, the random state is set as 42 with a minimum sample split number of two, and `gini’ is used as a criterion. Random forest was used as same as a Decision tree with a minimum of two split samples. On the other hand, SVM sets as a radial basis function (`RBF’) kernel. The learning rate was 0.1 with criterion `friedman_mse’ in GBM. However, the learning rate of LGBM was 0.05 with a bagging fraction of 0.8 and a bagging frequency of 5. A tree-based booster with a max depth of six was used in the XGB algorithm and the learning rate was 0.1.

To evaluate the machine learning models, a set of metrics are used, i.e., accuracy, precision, recall, f1-score, area under the receiver operating characteristic curve (ROC) curve (AUC), and log-losses. To find the associative parameters, we calculate the feature importance values for every machine learning model. The coefficient values of each feature represent the corresponding contribution of model training to separate an unknown instance among classes. The explanations of the following matrices are following:

- Accuracy: Accuracy can be determined in terms of positive and negative rates for binary classification, as seen below [31,32]:
  (1) $$Accuracy=\frac{TP+TN}{TP+TN+FP+FN}$$
  where *TP* = True Positives, *TN* = True Negatives, *FP* = False Positives, and *FN* = False Negatives.
- Precision: It determines the proportion of expected positives that actually materialize as positives. The True Positive (*TP*) and False Positive (*FP*) values are therefore important [31,33].
  (2) Precision=TP/(TP+FP)
- Recall: When we need to determine how many positives can be predicted, recall is another acceptable selection of assessment metric [31,32,33].
  (3) Recall=TP/(TP+FN)
- F1-Score: The F1-score maintains the balance between the classifier’s precision and recall. The F1 score, which is the consonant measure of precision & recall, is a value that falls between 0 and 1 [31,32].
  (4) $$F_{1}=2\times \left[\frac{Precision\times Recall}{Precision+Recall}\right]$$
- AUC: The area under the ROC curve, or AUC, shows how well the probabilities from the positive classes are separated from the probabilities from the negative classes. Where True Positive Rate, or TPR, is just the range of trues, we use it to figure out how many times a test is positive [31,32,33].
  (5) Sensitivity=TPR(TruePositiveRate)=Recall=TP/(TP+FN)
- Log-loss: The most important order metric based on probabilities is log loss. Raw log-loss values are hard to make sense of, but log-loss is a good way to measure models [31,32].
  (6) $$H_{p}\left(q\right)=-\frac{1}{N}\sum_{i=1}^{N}y_{i}\cdot log\left(p\left(y_{i}\right)\right)+\left(1-y_{i}\right)\cdot log\left(1-p\left(y_{i}\right)\right)$$

## 3. Results

In this study, we used two different types of factors with two different analyses and then correlated each of the results. The type of factors employed include features of the medical history of the patients who demonstrated reactions after vaccination, and the reaction natures were symptoms that arose after vaccination.

### 3.1. Distribution of Patient Medical History Features and Reactions

In this section, we describe the percentage of each significant factor of patient medical history and reactions shown in Table 1. Although the average age of the individuals was 47.5 years old, the age of those cases of fatalities and hospitalizations was 71.47 and 62.49 years, respectively. Thus, there is a clear difference in age between different patient groups. The highest number of people who received the first dose was 35.58%. For the second and third doses, these figures were 23.62% and 25.65%, respectively. In our study, there were approximately twice the number of female participants compared to male participants, and almost half of them were recorded as regularly taking other medications. A history of allergies (including various kinds of allergic events, not only anaphylaxis) was a frequently observed factor, with approximately 1 in 5 of the total cases and close to 1 in 4 of the fatality cases. In the hospitalized patient group, those with a history of allergies made up 1 in 4. In contrast, there were comparatively much fewer among SARS-CoV-2 positive patients, with 1 in 5. Other common diseases associated with significant patient reactions included *prior vaccine, type-2 diabetes, hypertension, thyroid disorder*, and *asthma* which each account for around 5%, while all the remaining factors each accounted for 1%–3%.

The reactions of patients are shown in Table 2. It can be seen that *chills* and *nausea* counts were 23.73% and 15.05%, respectively. In addition, *patient disability*, *headache and dyspnoea* count were around 10% of the total cases observed. The next most frequent adverse reactions include *pain in the extremity*, *pyrexia, fatigue, different kinds of pain, and dizziness* fall mainly in the range of 5% to 8%, with the incidence of other maladies below 5%. On the other hand, the lowest count was for *Anaphylactic reaction* (0.32%) and *Cardiac arrest* (0.35%), respectively.

### 3.2. Finding Significant Associations between Patient Medical History Factors and Post-Vaccination Adverse Reactions Using Statistical Analyses

**Figure 4:**
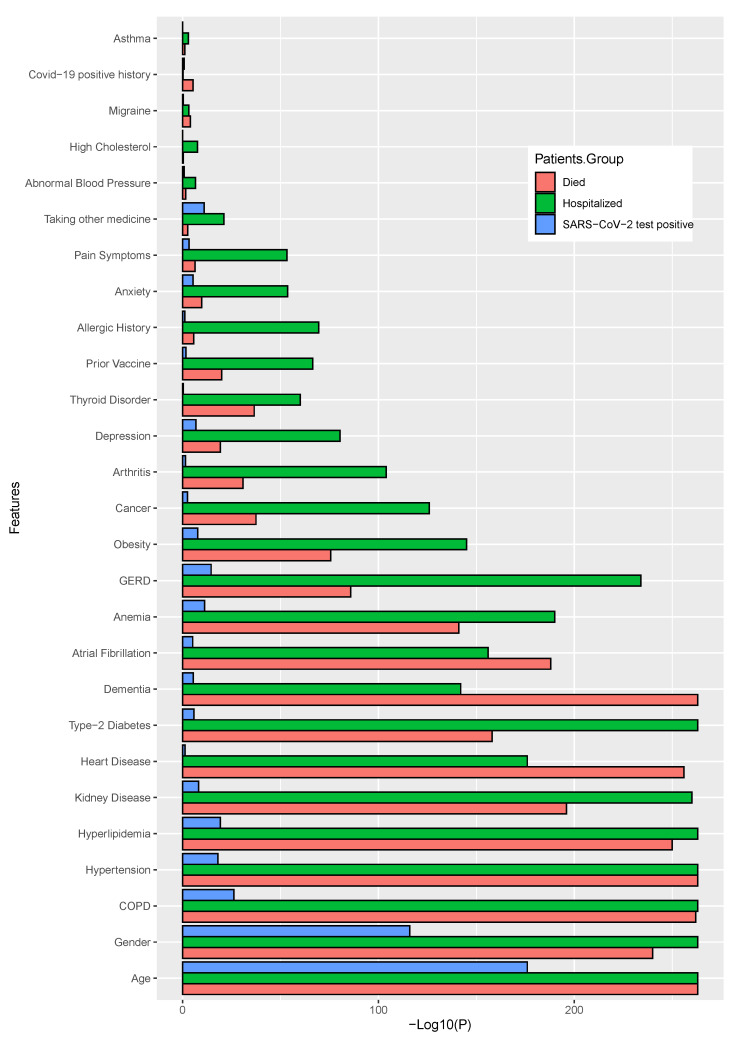
The significant features within the patients’ medical history, where the higher bar length indicates greater the significance.

**Figure 5:**
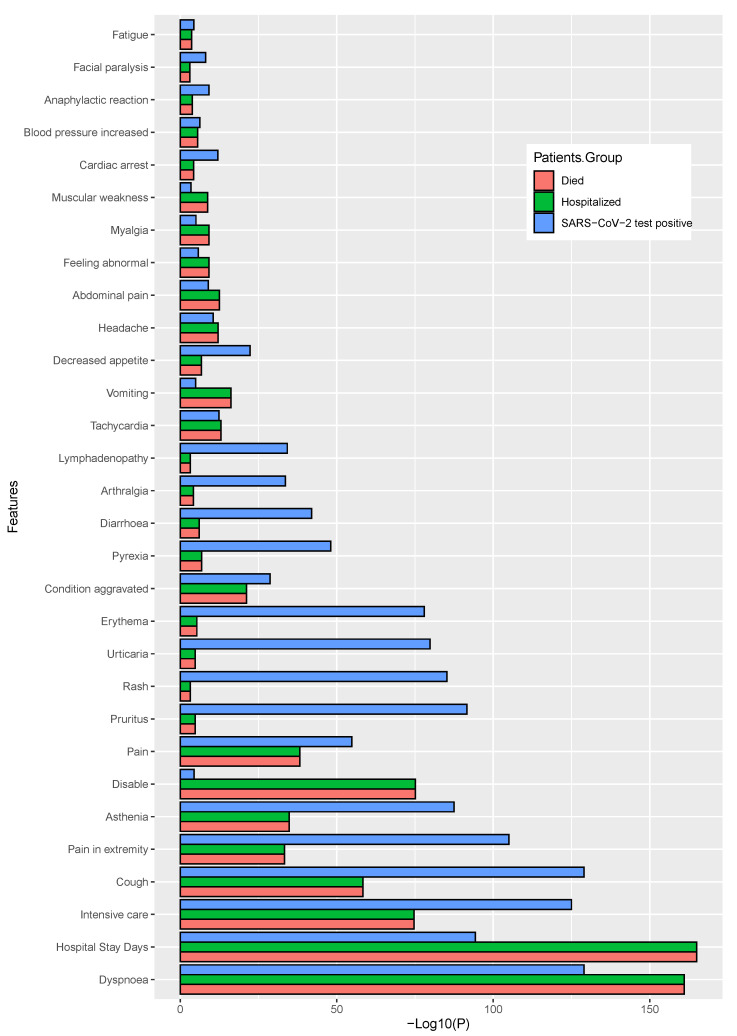
The significant patients’ adverse reactions after vaccination, where the higher bar length indicates greater the significance.

Using two different statistical tests (chi-squared test for categorical variables, Mann–Whitney U test for age variable), we identified the most associative and significant parameters, including patients’ medical history factors (including pre-existing diseases and other discomforts) and identified the adverse reactions or symptoms that may have predisposed to the development of severe health conditions, even fatality. In this analysis, we considered those significant parameters with a value of p<0.05 or lower. The target variables that we have used in our statistical analyses were *death*, *SARS-CoV-2 positive status*, and *hospital admission status*. The two different figures have (Figure 4 and Figure 5) been demonstrated. For a better view, we have calculated the negative 10-based logarithmic values for each of the *p* values and used them in the corresponding figures. Bar length indicates the significance level.

In terms of patients’ medical histories, *age, gender, COPD, hypertension, hyperlipidemia, kidney disease, heart disease* and *type-2 diabetes* were the most significant features among all the target groups. However, for patients’ death status, *dementia*, is also found as significant. Most of the significant parameters common for died, hospitalized and SARS-CoV-2 positive patients were *age, gender, COPD* and *hyperlipidemia*. However, *asthma, COVID-19 positive history, migraine*, and *high cholesterol* were not found as significant within any of the groups. The details results for this analysis are shown in Figure 4 and also shown the values in the Supplementary Table S7. In this figure, the bar lengths proportionate to the negative logarithm of *p*-values, while indicating the significance, i.e., a larger bar length is more significant.

We have also performed a similar analysis for the dataset with patient adverse reactions and identified a list of significantly associated symptoms (top 30) that are shown in Figure 5 and also shown the values in the Supplementary Table S8. In this case, we also considered three target variables as the independent variable, when it is not considered as a target variable at the time of analysis. It can be observed that the *dyspnoea, hospital stay duration in days, intensive care, cough*, and *pain in extremity* were the common factor for all three target variables. When we have considered the incidence of patient mortality as a target variable, *dyspnoea, hospital stay days, intensive care, cough*, and *disableness* were found to be the most significant. It was also observed that *dyspnoea, hospital stay days, intensive care, cough*, and *disability* were found as significant for the hospitalization status, whereas the *dyspnoea, cough, hospital stay days, intensive care, pruritus, rash, urticaria*, and *erythema* was for SARS-CoV-2 positive status.

### 3.3. Classification of Patients Using Machine Learning Algorithms

**Figure 6:**
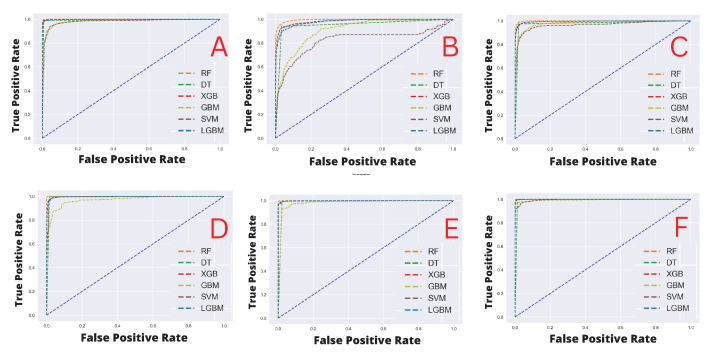
Area Under the ROC curves for the machine learning model evaluation. (**A**). classification of died patients’ using patients’ medical history dataset; (**B**). classification of SARS-CoV-2 positive patients’ using patients’ medical history dataset; (**C**). classification of hospitalised patients’ using patients’ medical history dataset; (**D**). classification of died patients’ using patients’ reaction dataset; (**E**). classification of SARS-CoV-2 positive patients’ using patients’ reaction dataset; (**F**). classification of hospitalized patients’ using patients’ reaction dataset.

In our machine learning analysis, first, we considered the patient medical histories as the independent features, and the *patient death*, *SARS-CoV-2 test positive*, and *hospital admission status* as dependent features, which depend on those independent features. Next, by considering both patient’s medical history and the patient reactions after vaccination. Initially, we trained our models and evaluated their performances with the test data by calculating a range of metrics including *accuracy, precision, recall, F1-score, ROC-AUC*, and *Log-loss*, which are shown in Table 3, including the ROC-AUC curves which are shown in Figure 6 in the panel’s A, B, and C, respectively for patients medical history. The results indicate that when the target feature variable was the patient’s death status, RF performed the best across all matrices, achieving the highest 1.0 scores and the lowest log-loss values (0.16). Other algorithms such as LGBM, DT, and XGB achieved an accuracy score of 0.99, whereas SVM and GBM achieved scores of 0.95 and 0.94, respectively. In terms of other metrics, the performances of all the algorithms were close to the accuracy scores. In addition, all of the methods achieve log loss values close to 2 percent. However, SVM and GBM model performances were encouraging, with above 94% accuracy. Similar observations were made when we considered *the SARS-CoV-2 test result* as the target feature variable, i.e., the RF outperformed other competing methods, with 0.96 accuracy scores, respectively, while other models’ performances were also found as competitive except SVM, which achieved almost consistently below 0.80, and the log-loss were also higher than others (i.e., above 7%). Finally, for the target variable, *hospital admission status*, RF, and DT have achieved the highest accuracy with 0.98% and all the other models performed almost equally, but scorewise, they have demonstrated some performances that are below optimal (i.e., compared to the previous two scenarios).

Next, we considered patient postvaccination adverse reactions as the independent feature, and the target variables remain the same as previously. The results indicating model predictive performances are shown in Table 4 including the ROC-AUC curves which are shown in Figure 6 in panels D, E, and F, respectively. It can be noted that all the classifiers demonstrated substantially similar performances with scores of greater than 0.80 in all the evaluation matrices and the log-loss was less than 3.50%. However, it can be also observed that when different target variables were set for the classification tasks after training with the patient adverse reaction, the best performing classifiers (in terms of Accuracy) were different as well, i.e., for the patient *death status*, the RF yielded a score of 1. Moreover, for the *SARS-CoV-2 test status*, and *hospital admission status*, the RF scored 1.0, and LGBM, DT, and XGB yielded 0.99 equally.

### 3.4. Feature Importance Analysis for Finding Significant Features Using Machine Learning Classifiers

**Figure 7:**
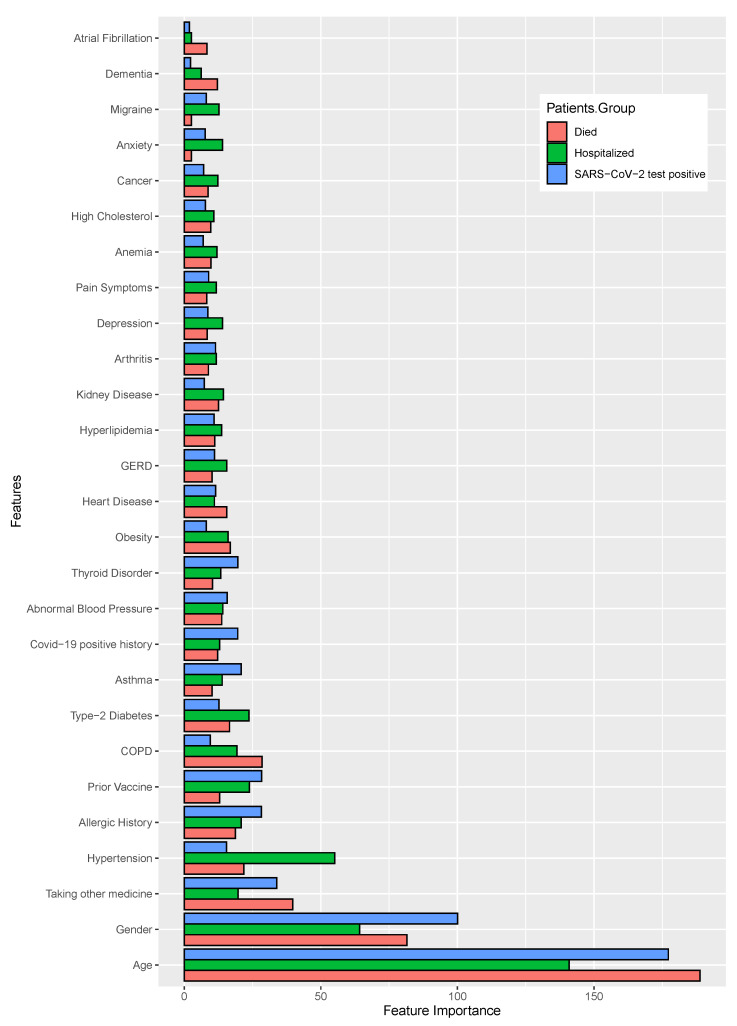
The features ranking according to the coefficient values of the patients’ medical history, calculated after machine learning model training. ML model outcomes indicate that higher coefficient values are mostly close to the significant association of severity.

**Figure 8:**
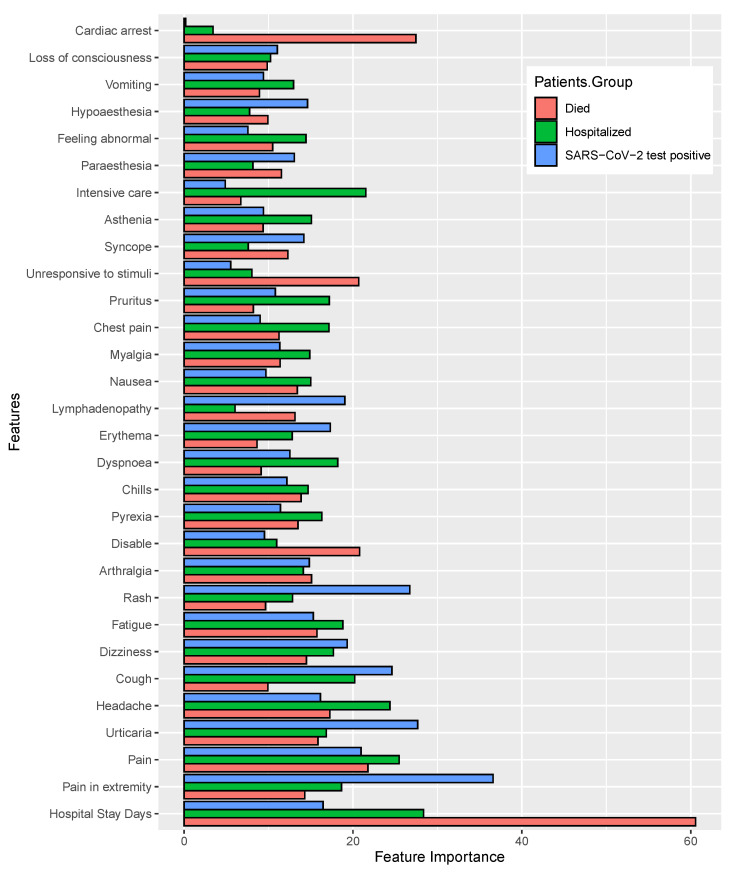
The features ranking according to the coefficient values of the patients’ adverse reactions, calculated after machine learning model training. ML model outcomes indicate that higher coefficient values are mostly close to the significant association of severity.

After model training, we calculated the coefficient values for each of the features and prioritized them as significant with regard to their corresponding target variables. Firstly, we calculated the feature importance scores for each distinct feature for individual machine learning classifiers (excluding SVM since it is not possible to find feature importance using the `RBF’ kernel), and then we normalized the values to render the data with the same scale, by using the quantile normalization technique [34]. This was followed by the average quantile normalization of those values, as shown in Figure 7 and Figure 8. The longest bar length indicates the higher rank of the features.

In the case of patient past medical histories, the identified features are shown in Figure 7 and also shown the values in the Supplementary Tables S1–S3, where the patient *age, gender*, and *taking other medicine* have shown significant importance for all target variable. With the target variables *death status* and *hospital admission status*, the important attributes were *hypertension* and *COPD*. *Allergic history* and *prior vaccine* showed the importance in case of both *SARS-CoV-2 positive status* and *hospital admission status*.

Figure 8 shows the feature importance of (top 30) listed according to the category of patient postvaccination adverse reactions or symptoms. Moreover, the coefficient values of each of the features are shown in Supplementary Tables S4–S6. For the first target variable (i.e., *patient mortality status*), the most important features identified were the *hospital treatment duration, severe pain, urticaria, headache, cough, dizziness, fatigue* and *rash*. For the second target variable (i.e., SARS-CoV-2 test status), the significant features were similar to the case of the first target variable, including that the *dyspnoea* was a novel finding as an important factor. Finally, for the third target variable, *hospital admission*, the significant features identified were *hospital treatment duration, fatigue, headache, dizziness, rash*, and *dyspnoea*.

## 4. Discussion

Vaccination is a well-accepted and reliable approach to prevent infectious diseases [35], and historically, it has proved to be one of the most effective strategies to control epidemics and pandemics, such as the SARS-CoV-2 outbreak [36]. All vaccines result in at least a small number of patients that demonstrate postvaccination side effects [37]. It is a challenging task to identify patients who are likely to show postvaccination adverse reactions. Some patients can experience a rapid onset reaction [23] requiring treatment at a hospital, or clinic, and even with rapidly administered care, the condition can be fatal [24]. Clearly, a better prediction of the risk of adverse reactions is highly desirable. If a model can distinguish between those whose health conditions pose a high risk and those who do not, hospital administration will be able to provide enough health care services. Therefore, this research could be quite facilitating for such cases.

The purpose of this research was to determine the key indications that indicate a susceptibility to adverse COVID-19 vaccination effects as well as to identify the key symptoms that indicate the cause or causes of the adverse conditions, including classifying a patient as at high risk or needs special care after COVID-19 vaccination. We have found a list of the most significant features that support our hypothesis; all of them are commonly found in all target groups. The most significant demographic information is patient *age* and *gender*; the most strongly associated patients’ coexisting conditions are *taking other medicine, hypertension, allergic history, and type-2 diabetes, abnormal blood pressure*; and most significant associated patient negative effects experienced postvaccination are *long time hospital treatment, pain, urticaria, headache, cough, dizziness*, and *rash*.

Furthermore, some postvaccination symptoms are commonly found in anonymous cases, but few of these are likely responsible for patients’ severe conditions due to vaccination. The most severe side effects identified are the *hospital treatment duration, headache, pyrexia, dyspnoea, chills, fatigue, different kind of pain*, and *dizziness*. The Centers for Disease Control and Prevention (CDC) reported headache, fatigue, soreness at the injection site, fever, and myalgias [38,39], which are similar to these early observations.

Patient allergic history is commonly an associated cause of adverse effects to many drugs and vaccines [34], and in the case of COVID-19, this has also been reported [40,41,42,43,44]; allergic-related reactions are found to a significant degree in every data group used in our study. Patient age is another important aspect, where the mortality rate in persons of advanced age is comparatively higher than in younger patients; previous studies have made similar findings [24]. There are reports that indicate allergic history may be a significant issue for COVID-19 vaccination [45,46]. However, our study indicates that patients taking significantly immunosuppressant medications [47] are at elevated risk of adverse reactions, as are those who are already SARS-CoV-2 positive at vaccination. Our research also suggests that other pre-existing conditions such as *COPD, hypertension, hyperlipidemia, kidney disease, type-2 diabetes*, and *heart disease* and a history of allergic responses could also be associated with the development of severe vaccine reactions. We also identified a range of other factors linked to significant patient reactions that require hospital treatments and may also be associated with patient mortality. In addition, the early findings demonstrate that some persons with chronic conditions, aging populations, or racial/ethnic minority populations require various types of vaccinations [39].

The utilization of machine learning models is widely acknowledged as capable of demonstrating morbidity/mortality-associated factor identification and for using those factors in making patient outcome predictions [21]. We identified machine learning models that performed well with our datasets and identified significant parameters related to vaccination-associated symptoms. The models achieved a good accuracy score including good precision, recall, and f1 score as well as low log loss values indicating strong classification and decision-making. In our analysis, we saw that several models performed particularly well with high scores for evaluation matrices, i.e., in the dataset of medical history, for the mortality and SARS-CoV-2 positive cases 1.0 accuracy for RF and for the hospitalized cases with 0.98 accuracy score for RF and DT, and in the dataset of adverse effects, for the mortality, SARS-CoV-2 positive, and hospitalized cases RF scored 1.0 accuracy score. Thus, based on the exhaustive comparison of various factors utilizing supervised machine learning models, this analysis may identify significant factors for clinicians, indicating parameters valuable for patient stratification. In sum, the use of machine learning models presented here to assess the likelihood that a patient is at risk of developing a severe reaction post-vaccination could be of great utility.

The limitation of this study is the availability of datasets. If we can collect the dataset for all other types of vaccines, we will be able to reach a definitive conclusion. However, this represents a groundbreaking contribution to the study of COVID-19 vaccination reactions.

Postvaccination adverse effects could be decreased if at-risk individuals can be identified based on the patient medical history, which this study confirms by experimenting with a set of validation datasets. Though vaccination may not be directly responsible for patients’ severe illness or death, we may need very careful observation of identified at-risk patients including access to ICU facilities.

## 5. Conclusions

The results indicate that patient medical histories are strongly related to the incidence of patient adverse reactions, some of which are associated with severe disease and even death. Moreover, a set of significant side effects are also developed as postvaccination symptoms. Therefore, it is important to identify possible causes of the adverse effects. If recognized, the factors identified can be taken into account by clinicians and enable care improvement.

Based on our analyses, the patients at greatest risk of adverse reaction after vaccination include those of advanced old age (Ageing—60 years or more), gender, COPD, hypertension, those having allergic conditions, those taking other medications (notably immunosuppressive medications) and those with a history of type-2 diabetes, hypertension or heart disease disorders. Moreover, the study also revealed that a set of symptoms postvaccination like *hospital stay duration, pyrexia, headache, dyspnoea, chills, fatigue, different kind of pain and dizziness, rash*, and *physical disability* are most associated with severe reactions.

Using statistical and machine learning analysis, we have found factors in patient medical histories that are associated with a risk or adverse patient reaction occurring in the postvaccination period. Our results also suggest that a common group of severe after-effects, that were identified by the independent analyses, proves that these outcomes are reliable.

Although our analysis reveals significant findings regarding the risk of COVID-19 vaccination effects, there are a few limitations that need further research effort. We have used a comparatively small amount of patient data collected from a specific region of the USA, which included those receiving the mRNA-based vaccines only. Therefore, for making a generalized decision, it is important to have a rigorous analysis with a larger population size and cover more vaccine types. Nevertheless, we hope that the result of this research will play a significant role for policymakers in considering the distribution of vaccines as well as identifying patients who may be vulnerable to adverse reactions.

The efficacy and safety of COVID-19 vaccines to date have been excellent considering they were so rapidly developed, but minor after-effects of an administered vaccine might be expected and some extreme allergic or other responses may infrequently happen. Although the possibility of postvaccination adverse effects is not always a reason to avoid vaccines (especially given the serious consequences of COVID-19 in many vulnerable groups), new information about adverse reaction risk that our study provides could be an important consideration in clinical considerations about how (or whether) to administer a COVID-19 vaccine to a possibly at-risk individual, as well as determining the need for extra monitoring and care at the point of vaccination.

## Figures and Tables

**Table 1:** The vaccination, demographic and patients’ medical history.

Patients’ Group	All Patients’	Died	SARS-CoV-2 Positive	Hospitalized
	n = 72,147	n = 2348	n = 13,546	n = 11,266
**Features**	**Count (%)**	**Count (%)**	**Count (%**)	**Count (%)**
**Vaccination Information**				
**Vaccine Dose**				
Dose 1	25,671 (35.58)	1032 (43.95)	4590 (33.88)	3960 (35.15)
Dose 2	17,039 (23.62)	445 (18.95)	2888 (21.32)	2518 (22.35)
Dose 3	18,505 (25.65)	535 (22.79)	3952 (29.17)	2998 (26.61)
Unknown	10,932 (15.15)	336 (14.31)	2116 (15.62)	1790 (15.89)
Days to Onset				
0	40,564 (56.22)	1128 (48.04)	6808 (50.26)	5692 (50.52)
>0	31,583 (43.78)	1220 (51.96)	6738 (49.74)	5574 (49.48)
**Demographic Information**				
Age (average) y	47.50	71.47	49.96	62.49
Gender				
Male	24,761 (34.32)	1247 (53.11)	5061 (37.36)	5270 (46.78)
Female	38,419 (53.25)	995 (42.38)	7700 (56.84)	5794 (51.43)
Unknown	8967 (12.43)	106 (4.51)	785 (5.8)	202 (1.79)
**Patients’ Medical History**				
Taking Other Medicine	33,733 (46.76)	1025 (43.65)	5976 (44.12)	4800 (42.61)
Prior Vaccine	3357 (4.65)	15 (0.64)	577 (4.26)	168 (1.49)
Allergic History	14,092 (19.53)	549 (23.38)	2722 (20.09)	2886 (25.62)
Type-2 Diabetes	2707 (3.75)	332 (14.14)	605 (4.47)	1233 (10.94)
Hypertension	3309 (4.59)	514 (21.89)	816 (6.02)	1761 (15.63)
Arthritis	1745 (2.42)	143 (6.09)	363 (2.68)	599 (5.32)
Asthma	2714 (3.76)	71 (3.02)	507 (3.74)	485 (4.3)
Migraine	985 (1.37)	10 (0.43)	174 (1.28)	193 (1.71)
High cholesterol	1085 (1.5)	40 (1.7)	205 (1.51)	236 (2.09)
Abnormal Blood Pressure	1606 (2.23)	36 (1.53)	279 (2.06)	176 (1.56)
COPD	1056 (1.46)	233 (9.92)	334 (2.47)	664 (5.89)
GERD	1340 (1.86)	171 (7.28)	364 (2.69)	640 (5.68)
Anxiety	1462 (2.03)	91 (3.88)	343 (2.53)	442 (3.92)
Obesity	1072 (1.49)	142 (6.05)	273 (2.02)	471 (4.18)
Depression	1357 (1.88)	104 (4.43)	330 (2.44)	465 (4.13)
Thyroid Disorder	2442 (3.38)	190 (8.09)	473 (3.49)	672 (5.96)
Anemia	704 (0.98)	142 (6.05)	204 (1.51)	393 (3.49)
Dementia	475 (0.66)	186 (7.92)	129 (0.95)	275 (2.44)
Cancer	1148 (1.59)	115 (4.9)	255 (1.88)	472 (4.19)
Kidney Disease	975 (1.35)	197 (8.39)	254 (1.88)	541 (4.8)
Hyperlipidemia	1379 (1.91)	266 (11.33)	391 (2.89)	835 (7.41)
Heart Disease	1501 (2.08)	282 (12.01)	311 (2.3)	629 (5.58)
COVID-19 Positive History	1184 (1.64)	67 (2.85)	242 (1.79)	176 (1.56)
Atrial Fibrillation	475 (0.66)	129 (5.49)	128 (0.94)	285 (2.53)
Pain Symptoms	1188 (1.65)	70 (2.98)	270 (1.99)	378 (3.36)

**Table 2:** The vaccination, demographic and patients’ medical history.

Patients’ Group	All Patients’	Died	SARS-CoV-2 Positive	Hospitalized
	n= 72,147	n = 2348	n = 13,546	n = 11,266
**Features**	**Count (%)**	**Count (%)**	**Count (%)**	**Count (%)**
Disable	7232 (10.02)	17 (0.72)	301 (2.22)	604 (5.36)
Headache	6545 (9.07)	139 (5.92)	1148 (8.47)	919 (8.16)
Pyrexia	4845 (6.72)	174 (7.41)	1672 (12.34)	1170 (10.39)
Dyspnoea	6851 (9.5)	263 (11.2)	2085 (15.39)	1419 (12.6)
Fatigue	4485 (6.22)	176 (7.5)	1412 (10.42)	964 (8.56)
Chills	17,117 (23.73)	71 (3.02)	775 (5.72)	648 (5.75)
Pain	4116 (5.71)	299 (12.73)	2513 (18.55)	2131 (18.92)
Dizziness	3964 (5.49)	80 (3.41)	395 (2.92)	630 (5.59)
Nausea	10,856 (15.05)	106 (4.51)	693 (5.12)	683 (6.06)
Pain in extremity	5403 (7.49)	87 (3.71)	410 (3.03)	531 (4.71)
Asthenia	2770 (3.84)	111 (4.73)	922 (6.81)	666 (5.91)
Vomiting	2198 (3.05)	88 (3.75)	492 (3.63)	484 (4.3)
Malaise	2199 (3.05)	86 (3.66)	641 (4.73)	380 (3.37)
Cough	3275 (4.54)	129 (5.49)	2192 (16.18)	841 (7.46)
Injection site pain	1677 (2.32)	42 (1.79)	53 (0.39)	234 (2.08)
Myalgia	2635 (3.65)	46 (1.96)	407 (3)	298 (2.65)
Hypoaesthesia	1731 (2.4)	33 (1.41)	106 (0.78)	292 (2.59)
Chest pain	2609 (3.62)	70 (2.98)	463 (3.42)	567 (5.03)
Feeling abnormal	2115 (2.93)	41 (1.75)	312 (2.3)	228 (2.02)
Rash	3343 (4.63)	72 (3.07)	194 (1.43)	451 (4)
Condition aggravated	2026 (2.81)	83 (3.53)	576 (4.25)	472 (4.19)
Chest discomfort	1342 (1.86)	28 (1.19)	260 (1.92)	275 (2.44)
Arthralgia	2706 (3.75)	48 (2.04)	264 (1.95)	348 (3.09)
Paraesthesia	1732 (2.4)	27 (1.15)	95 (0.7)	280 (2.49)
Unresponsive to stimuli	492 (0.68)	124 (5.28)	113 (0.83)	134 (1.19)
Diarrhoea	1697 (2.35)	51 (2.17)	537 (3.96)	338 (3)
Pruritus	3041 (4.22)	50 (2.13)	141 (1.04)	390 (3.46)
Heart rate increased	1123 (1.56)	17 (0.72)	102 (0.75)	187 (1.66)
Urticaria	2880 (3.99)	43 (1.83)	150 (1.11)	367 (3.26)
Facial paralysis	293 (0.41)	4 (0.17)	16 (0.12)	67 (0.59)
Syncope	1187 (1.65)	63 (2.68)	138 (1.02)	211 (1.87)
Tachycardia	858 (1.19)	29 (1.24)	244 (1.8)	213 (1.89)
Palpitations	1371 (1.9)	35 (1.49)	122 (0.9)	250 (2.22)
Hyperhidrosis	1082 (1.5)	22 (0.94)	112 (0.83)	162 (1.44)
Erythema	2859 (3.96)	63 (2.68)	152 (1.12)	359 (3.19)
Throat tightness	380 (0.53)	5 (0.21)	19 (0.14)	79 (0.7)
Tremor	927 (1.28)	27 (1.15)	98 (0.72)	149 (1.32)
Blood pressure increased	467 (0.65)	15 (0.64)	45 (0.33)	110 (0.98)
Anaphylactic reaction	228 (0.32)	7 (0.3)	6 (0.04)	57 (0.51)
Intensive care	556 (0.77)	65 (2.77)	324 (2.39)	244 (2.17)
Loss of consciousness	700 (0.97)	28 (1.19)	60 (0.44)	129 (1.15)
Decreased appetite	902 (1.25)	49 (2.09)	285 (2.1)	198 (1.76)
Muscular weakness	709 (0.98)	20 (0.85)	96 (0.71)	169 (1.5)
Flushing	374 (0.52)	4 (0.17)	17 (0.13)	57 (0.51)
Mobility decreased	1135 (1.57)	24 (1.02)	116 (0.86)	201 (1.78)
Injection site erythema	767 (1.06)	13 (0.55)	19 (0.14)	119 (1.06)
Feeling hot	615 (0.85)	11 (0.47)	39 (0.29)	79 (0.7)
Abdominal pain	1311 (1.82)	36 (1.53)	332 (2.45)	300 (2.66)
Injection site swelling	775 (1.07)	17 (0.72)	19 (0.14)	114 (1.01)
Cerebrovascular accident	401 (0.56)	31 (1.32)	70 (0.52)	155 (1.38)
Cardiac arrest	256 (0.35)	102 (4.34)	93 (0.69)	64 (0.57)
Lymphadenopathy	1876 (2.6)	29 (1.24)	146 (1.08)	239 (2.12)

**Table 3:** Comparative performance evaluation for the patient classification based on machine learning algorithms with the dataset of patients’ medical history.

Target Variable	Model	Accuracy	Precision	Recall	F1-Score	AUC	Log Loss
Died	RF	1.0	1.0	1.0	1.0	1.0	0.16
	LGBM	0.99	0.99	0.98	0.99	0.99	0.47
	SVM	0.95	0.95	0.93	0.94	0.95	1.84
	DT	0.99	0.99	0.99	0.99	0.99	0.28
	XGB	0.99	0.99	0.99	0.99	0.99	0.35
	GBM	0.94	0.94	0.93	0.93	0.94	2.13
SARS CoV-2 Positive	RF	0.96	0.96	0.96	0.96	0.96	1.22
	LGBM	0.93	0.94	0.91	0.92	0.93	2.36
	SVM	0.78	0.82	0.67	0.74	0.77	7.54
	DT	0.95	0.96	0.94	0.95	0.95	1.61
	XGB	0.95	0.95	0.93	0.94	0.95	1.83
	GBM	0.82	0.81	0.79	0.8	0.82	6.15
Hospitalized	RF	0.98	0.98	0.98	0.98	0.98	0.6
	LGBM	0.97	0.97	0.97	0.97	0.97	1.04
	SVM	0.92	0.94	0.9	0.92	0.92	2.6
	DT	0.98	0.98	0.97	0.98	0.98	0.79
	XGB	0.97	0.98	0.97	0.97	0.97	0.95
	GBM	0.93	0.94	0.9	0.92	0.93	2.47

**Table 4:** Comparative performance evaluation for the patient classification based on machine learning algorithms with the dataset of patients’ adverse reactions after vaccination.

Target Variable	Model	Accuracy	Precision	Recall	F1-Score	AUC	Log Loss
Died	RF	1.0	1.0	1.0	1.0	1.0	0.09
	LGBM	0.98	0.98	0.98	0.98	0.98	0.84
	SVM	0.98	0.98	0.98	0.98	0.98	0.74
	DT	0.99	0.99	1	0.99	0.99	0.28
	XGB	0.98	0.98	0.98	0.98	0.98	0.68
	GBM	0.91	0.95	0.88	0.92	0.91	3.08
SARS-CoV-2 Positive	RF	1.0	1.0	1.0	1.0	1.0	0.11
	LGBM	0.99	0.99	0.99	0.99	0.98	0.47
	SVM	0.99	0.99	0.99	0.99	0.98	0.52
	DT	0.99	0.99	1.0	0.99	0.99	0.34
	XGB	0.99	0.99	0.99	0.99	0.99	0.41
	GBM	0.93	0.92	0.99	0.95	0.9	2.33
Hospitalized	RF	1.0	1.0	1.0	1.0	1.0	0.07
	LGBM	0.99	1.0	0.99	0.99	0.99	0.27
	SVM	0.96	0.98	0.96	0.97	0.96	1.34
	DT	0.99	0.99	1.0	1.0	0.99	0.19
	XGB	0.99	0.99	0.99	0.99	0.99	0.25
	GBM	0.97	1.0	0.96	0.98	0.98	0.97

## Data Availability

The dataset and corresponding codes are available on the following repositories: Dataset: https://vaers.hhs.gov/data/datasets.html (accessed on 16 February 2022), Codes: https://github.com/m-moni/COVID-19 (accessed on 1 October 2022).

## Supplementary Materials

The following supporting information can be downloaded at: https://www.mdpi.com/article/10.3390/healthcare11010031/s1, Table S1: Coefficient values of the patient’s medical history after machine learning model training for target variable Died. Table S2: Coefficient values of the patient’s medical history after machine learning model training for target variable SARS-CoV-2 Positive. Table S3: Coefficient values of the patient’s medical history after machine learning model training for target variable Hospitalized. Table S4: Coefficient values of the patient’s reactions after machine learning model training for target variable Died. Table S5: Coefficient values of the patient’s reactions after machine learning model training for target variable SARS-CoV-2 Positive. Table S6: Coefficient values of the patient’s reactions after machine learning model training for target variable Hospitalized. Table S7: *p*-values for the patient’s medical history of statistical analysis. Table S8: *p*-values for the patient’s reaction of statistical analysis.

## Abbreviations

The following abbreviations are used in this manuscript:

SARS-CoV-2	Severe Acute Respiratory Syndrome Coronavirus 2
COVID-19	Corona virus disease 2019
MERS	Middle East respiratory syndrome
VAERS	Vaccine Adverse Event Reporting System
COPD	Chronic obstructive pulmonary disease
DT	Decision Tree
RF	Random Forest
SVM	Support Vector Machine
GBM	Gradient Boosting Machine
XGB	Extreme Gradient Boosting Machine
LGBM	Light Gradient Boosting Machine
