# Supplementary material for: Adverse Effects of COVID-19 Vaccination: Machine Learning and Statistical Approach to Identify and Classify Incidences of Morbidity and Postvaccination Reactogenicity
Source: Healthcare (Basel). 2022 Dec 22;11(1):31. doi: 10.3390/healthcare11010031 (PMC9819062; doi:10.3390/healthcare11010031)
Supplement: Supplementary file 1 [file healthcare-11-00031-s001.zip › healthcare-1996165-supplementary.pdf]

Supplementary Table S1: Coefficient values of the patient's medical history after machine learning model training for target variable Died

Features	RF	LGBM	DT	XGB	GBM
Age	0.485771	942	0.6452226	0.1245845	0.5455121
Gender	0.1675705	407	0.1367884	0.1093783	0.1631078
Taking other medicine	0.0545965	220	0.0475745	0.0673434	0.0350146
Prior Vaccine	0.0091065	67	0.0030951	0.0509157	0.0145328
Allergic History	0.0244581	143	0.0179043	0.0414204	0.0101271
Type-2 Diabetes	0.0227197	112	0.0119516	0.0312908	0.0179126
Hypertension	0.0447308	79	0.0179856	0.0367234	0.0390901
Arthritis	0.0041013	43	0.0049471	0.0233567	0.000932
Asthma	0.0067543	49	0.005158	0.0261796	0.0036572
Migraine	0.0013154	13	0.0006978	0.0086436	0.0006018
High Cholesterol	0.0049735	49	0.0035152	0.0221677	0.0046422
Abnormal Blood Pressure	0.00785	65	0.0060055	0.0529463	0.0067236
COPD	0.0312861	93	0.0226397	0.0566736	0.0419738
GERD	0.0091695	53	0.003867	0.0201832	0.0042683
Anxiety	0.002163	13	0.0010229	0.0051668	6.13E-05
Obesity	0.0145271	94	0.0179046	0.0497325	0.0168409
Depression	0.0033758	38	0.0038322	0.0225264	0.0012705
Thyroid Disorder	0.0064078	53	0.0061626	0.0253098	0.0010826
Anemia	0.0048687	42	0.0074102	0.0246055	0.0019226
Dementia	0.0172876	50	0.0024169	0.0254867	0.0200888
Cancer	0.0045387	43	0.0026994	0.0212076	0.0036328
Kidney Disease	0.0112692	60	0.0057147	0.0320093	0.0167698
Hyperlipidemia	0.0206609	50	0.0028132	0.0234155	0.0141904
Heart Disease	0.0264228	68	0.0091028	0.0307607	0.0300771
Covid-19 positive history	0.0062515	73	0.008084	0.038479	0.0017898
Atrial Fibrillation	0.0041862	40	0.0023417	0.0141483	0.0024952
Pain Symptoms	0.0036374	41	0.0031425	0.0153447	0.001682

Supplementary Table S2: Coefficient values of the patient's medical history after machine learning model training for target variable SARS-CoV-2 Positive

**Features**	RF	LGBM	DT	XGB	GBM
Age	0.5031024	1013	0.4848036	0.0718361	0.4794835
Gender	0.1448509	371	0.1407043	0.0790358	0.2039688
Taking other medicine	0.0343001	183	0.0474763	0.0535841	0.0464872
Prior Vaccine	0.0277164	116	0.0332161	0.0537374	0.0291541
Allergic History	0.0332022	180	0.0498366	0.0315843	0.0283585
Type-2 Diabetes	0.0163823	65	0.0167252	0.0292931	0.0156867
Hypertension	0.0235027	71	0.0170618	0.0328357	0.0193941
Arthritis	0.0123351	53	0.0112209	0.0371482	0.00664
Asthma	0.0243393	105	0.0268061	0.0493217	0.022032
Migraine	0.007955	50	0.007508	0.0294603	0.00418
High Cholesterol	0.0087637	33	0.0094759	0.0292113	0.0053496
Abnormal Blood Pressure	0.0173137	72	0.0175617	0.0502221	0.0178796
COPD	0.0105335	36	0.0058998	0.0341863	0.0163968
GERD	0.010629	49	0.008611	0.0374572	0.0142622
Anxiety	0.0087206	36	0.0090865	0.020828	0.005587
Obesity	0.0090663	44	0.0082768	0.0267975	0.0062914
Depression	0.0094441	34	0.0090157	0.0312226	0.0112459
Thyroid Disorder	0.0201361	100	0.0221342	0.0439322	0.0211008
Anemia	0.0052753	37	0.006475	0.0240324	0.0035913
Dementia	0.0030516	12	0.0034611	0.0160027	0.000593
Cancer	0.0087461	36	0.00671	0.0253861	0.0022681
Kidney Disease	0.0076138	43	0.0080313	0.0219994	0.003952
Hyperlipidemia	0.0139955	41	0.0087125	0.0368747	0.0135405
Heart Disease	0.010827	70	0.0097856	0.0355662	0.0089541
Covid-19 positive history	0.0158452	95	0.0188252	0.0549697	0.0082607
Atrial Fibrillation	0.0031065	9	0.0033236	0.0112322	0.0001634
Pain Symptoms	0.0092457	46	0.009255	0.0322427	0.0051788

Supplementary Table S3: Coefficient values of the patient's medical history after machine learning model training for target variable Hospitalized

**Features**	RF	LGBM	DT	XGB	GBM
Age	0.4659203	785	0.5529693	0.0795364	0.539623
Gender	0.1415945	374	0.111549	0.0564173	0.13729
Taking other medicine	0.0279721	161	0.0181093	0.0281842	0.016261
Prior Vaccine	0.0245912	92	0.0372044	0.0705576	0.0283945
Allergic History	0.0336667	149	0.0311539	0.0398741	0.0178627
Type-2 Diabetes	0.0408495	101	0.0292529	0.0473447	0.0457905
Hypertension	0.0670752	107	0.0712576	0.1062071	0.0703976
Arthritis	0.0070962	51	0.0068206	0.0198634	0.0020683
Asthma	0.0109516	69	0.0091428	0.0332873	0.0030911
Migraine	0.0068353	60	0.0085569	0.0280571	0.0044619
High Cholesterol	0.0056054	54	0.005922	0.0127428	0.0013421
Abnormal Blood Pressure	0.011027	65	0.0108181	0.030553	0.0083601
COPD	0.0230241	68	0.0144266	0.0664303	0.0320645
GERD	0.0160587	68	0.0112016	0.0355828	0.0224099
Anxiety	0.0090031	72	0.0105052	0.0338302	0.00572
Obesity	0.0113968	80	0.0094128	0.0477429	0.0098182
Depression	0.0096938	74	0.0083476	0.03487	0.0061815
Thyroid Disorder	0.0104579	73	0.0090196	0.0302862	0.0016401
Anemia	0.0062806	60	0.0046143	0.031536	0.0036639
Dementia	0.0036156	26	0.0020225	0.0085986	0.0011769
Cancer	0.0080139	61	0.0064022	0.0183649	0.0044396
Kidney Disease	0.010807	75	0.0070791	0.0389908	0.0130753
Hyperlipidemia	0.024026	62	0.0040508	0.0325605	0.0205371
Heart Disease	0.0098778	63	0.0050901	0.0194216	0.0003338
Covid-19 positive history	0.0065354	78	0.0074179	0.0261971	0.0017782
Atrial Fibrillation	0.0016764	13	0.0013519	0.0050953	1.84E-05
Pain Symptoms	0.0063477	59	0.0063012	0.0178678	0.0021997

Supplementary Table S4: Coefficient values of the patient's reactions after machine learning model training for target variable Died

Features	RF	LGBM	DT	XGB	GBM
Hospital Stay Days	0.1903703	346	0.3475123	0.041893	0.355557
Disable	0.0416797	93	0.0244903	0.0416867	0.0537616
Headache	0.0452953	111	0.0143977	0.0127466	0.0352031
Pyrexia	0.0197745	126	0.0262676	0.0064219	0.0003904
Dyspnoea	0.0148237	79	0.0148488	0.0041827	0.0004468
Fatigue	0.0303269	97	0.0301042	0.0078205	0.0143229
Chills	0.032794	45	0.0103741	0.0201617	0.0319619
Pain	0.1106506	129	0.0326766	0.0147198	0.1134494
Dizziness	0.0254569	63	0.0277695	0.0164378	0.0230102
Nausea	0.0241016	97	0.015461	0.0129678	0.0142858
Pain in extremity	0.025879	100	0.0222244	0.0122159	0.0049445
Asthenia	0.0131383	58	0.0093871	0.0121451	0.0068366
Vomiting	0.0104165	86	0.0119724	0.005617	0.0002906
Malaise	0.0092769	45	0.0074977	0.0087954	0.0019401
Cough	0.0139219	69	0.0194309	0.0061933	0.0003379
Injection site pain	0.0057959	23	0.0066554	0.006071	0.0008008
Myalgia	0.0172028	62	0.0096872	0.0152721	0.0202019
Hypoaesthesia	0.0108674	39	0.0073745	0.0228748	0.0065187
Chest pain	0.0135525	93	0.0156423	0.0113201	0.0023431
Feeling abnormal	0.009821	38	0.008313	0.0297487	0.0080927
Rash	0.0158605	48	0.0071569	0.015304	0.0123756
Condition aggravated	0.0083364	41	0.0191574	0.0058287	0.0005089
Chest discomfort	0.0083001	34	0.0051959	0.02178	0.0080591
Arthralgia	0.0267916	49	0.0098278	0.0371636	0.0267478
Paraesthesia	0.0159687	45	0.004918	0.0301733	0.0138342
Unresponsive to stimuli	0.0313612	35	0.08305	0.1077292	0.0723123
Diarrhoea	0.0092694	38	0.0099459	0.0044498	0.0016297
Pruritus	0.0103272	35	0.0053515	0.0160232	0.0071925
Heart rate increased	0.0130296	43	0.0054152	0.0246357	0.0091978

Urticaria	0.0356855	54	0.0107984	0.0393831	0.0387532
Facial paralysis	0.0047323	32	0.0004634	0.0149116	0.0031853
Syncope	0.0113586	74	0.0245817	0.0165097	0.0047024
Tachycardia	0.0051666	29	0.0037222	0.0086795	0.001124
Palpitations	0.0099865	48	0.0062424	0.0094562	0.0062374
Hyperhidrosis	0.0075586	39	0.0048995	0.0185066	0.0051158
Erythema	0.0119586	35	0.0085241	0.010247	0.0098973
Throat tightness	0.003522	29	0.0009619	0.0115257	0.0014969
Tremor	0.0020653	18	0.0016465	0.0051084	5.98E-05
Blood pressure increased	0.0021275	14	0.0019292	0.0042077	2.42E-10
Anaphylactic reaction	0.0031129	28	0.0001953	0.0106264	0.0016856
Intensive care	0.0040045	45	0.0072163	0.0085449	0.0001251
Loss of consciousness	0.0102666	42	0.0064619	0.0223929	0.0083739
Decreased appetite	0.0077454	40	0.0177556	0.0059726	0.0004035
Muscular weakness	0.0018982	15	0.0005585	0.0126424	0.0004206
Flushing	0.0019723	22	0.000836	0.0053028	0.0010136
Mobility decreased	0.0059498	42	0.0057817	0.0156649	0.0029296
Injection site erythema	0.0046106	23	0.0035957	0.0190568	0.0019645
Feeling hot	0.0036999	26	0.0019961	0.0103446	0.0006944
Abdominal pain	0.0060206	40	0.0064547	0.0079246	0.000669
Injection site swelling	0.0047753	43	0.0013845	0.0121895	0.000793
Cerebrovascular accident	0.0018206	17	0.0026256	0.006518	9.95E-06
Cardiac arrest	0.0255325	34	0.0642041	0.1109572	0.0390972
Lymphadenopathy	0.020039	44	0.0050593	0.0409477	0.0246942

Supplementary Table S5: Coefficient values of the patient's reactions after machine learning model training for target variable SARS-CoV-2 Positive

Features	RF	LGBM	DT	XGB	GBM
Hospital Stay Days	0.02429	229	0.018705	0.004737	0.0001559
Disable	0.00586	43	0.0047771	0.0186074	0.0060365
Headache	0.03986	136	0.0234414	0.0155864	0.0260355

Pyrexia	0.01265	110	0.0145	0.003798	0.0011235
Dyspnoea	0.02188	90	0.0211586	0.0050748	0.0201007
Fatigue	0.02292	162	0.0180316	0.0085624	0.0060807
Chills	0.01598	94	0.013013	0.0099961	0.0094973
Pain	0.05858	150	0.0130282	0.0159628	0.0935004
Dizziness	0.05109	61	0.0704763	0.0351982	0.0721778
Nausea	0.01018	68	0.0075318	0.0050794	0.000893
Pain in extremity	0.08514	64	0.1388041	0.0749193	0.1104952
Asthenia	0.01211	64	0.0121799	0.0057585	0.0003797
Vomiting	0.00954	68	0.0076787	0.0075908	0.0002919
Malaise	0.00491	48	0.0041958	0.002784	0.0005362
Cough	0.07991	106	0.0892575	0.0404813	0.0692667
Injection site pain	0.02347	57	0.0320557	0.0308696	0.0216211
Myalgia	0.01644	67	0.0122118	0.0146521	0.0132853
Hypoaesthesia	0.02785	46	0.0367351	0.0447536	0.0380497
Chest pain	0.0069	76	0.005974	0.0031296	0.0007596
Feeling abnormal	0.00523	41	0.0043066	0.0036289	0.0018389
Rash	0.06549	56	0.0700644	0.0819563	0.0893059
Condition aggravated	0.00302	23	0.0019546	0.0022993	0.0001535
Chest discomfort	0.00617	50	0.0038683	0.006863	0.0026881
Arthralgia	0.03497	69	0.0395472	0.023979	0.0482764
Paraesthesia	0.02813	49	0.0233114	0.0390566	0.0256564
Unresponsive to stimuli	0.00148	22	0.000823	0.0094224	0.000337
Diarrhoea	0.00613	51	0.0037677	0.0043507	0.0006596
Pruritus	0.02588	52	0.0019437	0.0150777	0.0320001
Heart rate increased	0.01006	37	0.0093696	0.0216841	0.0111617
Urticaria	0.07675	66	0.0956275	0.0746705	0.0965025
Facial paralysis	0.00166	19	0.0007136	0.0061165	0.0004446
Syncope	0.02758	41	0.0262782	0.0525882	0.032473
Tachycardia	0.00123	8	0.0005035	0.0029566	3.29E-05
Palpitations	0.01349	51	0.0161454	0.0098082	0.0137491
Hyperhidrosis	0.00609	38	0.0016526	0.0091826	0.0018836

Erythema	0.04316	56	0.0494311	0.0431256	0.0573969
Throat tightness	0.001	30	0.0004688	0.0041164	0.0002101
Tremor	0.01141	46	0.0109377	0.029027	0.0105478
Blood pressure increased	0.00586	36	0.0049054	0.0190234	0.0044525
Anaphylactic reaction	0.00581	44	0.0061079	0.0158962	0.0037329
Intensive care	0.00161	20	0.0019739	0.0034721	0.0001371
Loss of consciousness	0.01363	38	0.0132857	0.0345504	0.0140706
Decreased appetite	0.00136	8	0.0005827	0.001236	0.0001006
Muscular weakness	0.00199	25	0.0015437	0.0027713	3.18E-07
Flushing	0.00178	26	0.0010274	0.0066711	5.36E-05
Mobility decreased	0.00324	31	0.0010822	0.01187	0.0007659
Injection site erythema	0.00449	29	0.0012876	0.0067546	0.0019953
Feeling hot	0.00396	40	0.0027983	0.0121516	0.0015767
Abdominal pain	0.00876	45	0.0022915	0.0091265	0.0016414
Injection site swelling	0.0073	40	0.0062185	0.0139018	0.0025121
Cerebrovascular accident	0.00133	20	0.0016162	0.0065091	0.0002716
Cardiac arrest	0.00042	1	0.000337	0.0003451	0
Lymphadenopathy	0.03998	53	0.0504716	0.0682699	0.0530847

Supplementary Table S6: Coefficient values of the patient's reactions after machine learning model training for target variable Hospitalized

Features	RF	LGBM	DT	XGB	GBM
Hospital Stay Days	0.6687034	92	0.8551265	0.85527	0.8562615
Disable	0.0032561	54	0.0026147	0.0030694	0.0005839
Headache	0.0246609	141	0.0081884	0.0033423	0.0149533
Pyrexia	0.0072799	123	0.0048537	0.0020887	0.0012887
Dyspnoea	0.0105327	87	0.006728	0.0036965	0.0072617
Fatigue	0.0194094	140	0.0068564	0.0020075	0.0041531
Chills	0.0091291	94	0.0042592	0.0021964	0.0007499
Pain	0.0547878	151	0.0117751	0.002796	0.0318569

Dizziness	0.0147373	101	0.0043483	0.0026871	0.008703
Nausea	0.0068278	104	0.0040819	0.0021364	0.0024523
Pain in extremity	0.0212071	78	0.0028998	0.0035341	0.0115743
Asthenia	0.0062028	103	0.0040037	0.002908	0.001243
Vomiting	0.0051188	87	0.0027095	0.0027048	0.0010821
Malaise	0.0045278	66	0.0035715	0.0027538	0.000275
Cough	0.0116455	85	0.01132	0.0056241	0.0068697
Injection site pain	0.0031832	37	0.001911	0.0028845	6.52E-05
Myalgia	0.00925	59	0.0026346	0.0023493	0.013831
Hypoaesthesia	0.0025748	57	0.000969	0.0017915	6.67E-05
Chest pain	0.008336	86	0.0038228	0.0040397	0.0041367
Feeling abnormal	0.008847	44	0.0022415	0.0040311	0.0059351
Rash	0.0056914	76	0.0021419	0.0038277	0.0006833
Condition aggravated	0.0048675	65	0.0042118	0.0049347	0.0005142
Chest discomfort	0.0018867	30	0.0009345	0.0012953	0.0001432
Arthralgia	0.0097182	82	0.0023385	0.0021607	0.0040526
Paraesthesia	0.0032082	44	0.0016921	0.0021377	8.69E-05
Unresponsive to stimuli	0.0013914	13	0.0020047	0.0046616	2.50E-05
Diarrhoea	0.0034889	31	0.0021744	0.0011097	1.28E-05
Pruritus	0.0063221	84	0.0046143	0.0057221	0.0012431
Heart rate increased	0.0030004	33	0.001242	0.0014323	0.0006845
Urticaria	0.0105858	45	0.0023086	0.0060186	0.0043937
Facial paralysis	0.0003175	1	0.0003316	0.0004371	1.81E-06
Syncope	0.00287	34	0.0009123	0.0025751	0.0002753
Tachycardia	0.0016531	32	0.0010562	0.0013642	8.35E-05
Palpitations	0.0023352	38	0.001528	0.0032854	0.0004344
Hyperhidrosis	0.0023346	36	0.0010139	0.0016319	0.001359
Erythema	0.0070281	42	0.0027882	0.0030867	0.0012304
Throat tightness	0.0012839	27	0.0006308	0.0014705	2.71E-05
Tremor	0.0019958	59	0.0013914	0.0018026	0.0004675
Blood pressure increased	0.0008944	19	0.0002199	0.0011753	1.26E-05

Anaphylactic reaction	0.0002148	15	0.0002536	0.0012312	0
Intensive care	0.0106942	60	0.0114348	0.0143812	0.0093363
Loss of consciousness	0.0023901	50	0.0011929	0.0038017	0.0005167
Decreased appetite	0.0015689	22	0.0007964	0.000838	1.51E-05
Muscular weakness	0.001254	41	0.0007133	0.0028752	6.73E-06
Flushing	0.0008362	18	0.0001626	0.0010853	0.0001288
Mobility decreased	0.0015981	31	0.0007234	0.0013362	0.0004741
Injection site erythema	0.0011241	32	0.0009071	0.0017423	6.92E-05
Feeling hot	0.0019689	19	0.0007015	0.0011408	6.28E-05
Abdominal pain	0.0020432	37	0.0011975	0.0026802	0.0001006
Injection site swelling	0.0017676	40	0.0009471	0.0033041	8.53E-05
Cerebrovascular accident	0.0009411	22	0.0006947	0.0009202	2.41E-05
Cardiac arrest	0.000421	12	0.0003287	0.0011098	6.07E-05
Lymphadenopathy	0.0020871	21	0.0014958	0.0015135	4.55E-05

Supplementary Table S7: P-values for the patient's medical history of statistical analysis

**Features**	Died	SARS-CoV-2 test positive	Hospitalized
Age	0	1.56E-176	0
Gender	8.93E-241	3.51E-117	0
Taking other medicine	0.0023536	8.95E-12	8.00E-22
Prior Vaccine	9.73E-21	0.016866254	3.31E-67
Allergic History	1.97E-06	0.068890892	2.91E-70
Type-2 Diabetes	4.45E-159	1.38E-06	0
Hypertension	0	8.67E-19	0
Arthritis	1.19E-31	0.030490758	5.05E-105
Asthma	0.06353	0.917450232	0.001068276
Migraine	9.72E-05	0.39110191	0.000627669
High Cholesterol	0.4701951	0.950954946	2.58E-08
Abnormal Blood Pressure	0.0249393	0.154458671	2.42E-07

COPD	1.48E-262	6.97E-27	0
GERD	1.47E-86	2.74E-15	2.63E-234
Anxiety	1.65E-10	4.21E-06	2.59E-54
Obesity	2.55E-76	1.99E-08	1.35E-145
Depression	4.98E-20	1.58E-07	4.45E-81
Thyroid Disorder	2.55E-37	0.460447051	7.45E-61
Anemia	2.34E-141	4.61E-12	4.88E-191
Dementia	0	3.58E-06	2.22E-142
Cancer	2.90E-38	0.002998129	8.85E-127
Kidney Disease	5.69E-197	6.03E-09	1.25E-260
Hyperlipidemia	1.58E-250	5.11E-20	0
Heart Disease	2.43E-256	0.055419916	1.98E-176
Covid-19 positive history	3.86E-06	0.149719229	0.498448314
Atrial Fibrillation	4.68E-189	6.28E-06	9.63E-157
Pain Symptoms	3.69E-07	0.000502461	5.30E-54

Supplementary Table S8: P-values for the patient's reaction of statistical analysis

Features	Died	SARS-CoV-2 test positive	Hospitalized
Hospital Stay Days	0	4.88E-95	0
Disable	7.09E-76	3.63E-05	7.09E-76
Headache	7.79E-13	3.02E-11	7.79E-13
Pyrexia	1.39E-07	7.16E-49	1.39E-07
Dyspnoea	5.10E-162	0	5.10E-162
Fatigue	0.0002295	4.68E-05	0.000229457
Chills	0.0276427	0.00856473	0.027642737
Pain	6.16E-39	1.65E-55	6.16E-39
Dizziness	0.5887235	3.04E-54	0.588723466
Nausea	0.0042577	0.033687561	0.004257706
Pain in extremity	4.78E-34	4.53E-106	4.78E-34
Asthenia	1.70E-35	2.92E-88	1.70E-35

Vomiting	5.71E-17	1.23E-05	5.71E-17
Malaise	0.0311674	1.56E-36	0.0311674
Cough	3.99E-59	0	3.99E-59
Injection site pain	0.0624723	2.00E-61	0.062472348
Myalgia	6.57E-10	9.27E-06	6.57E-10
Hypoaesthesia	0.1553858	3.36E-42	0.155385773
Chest pain	2.33E-18	0.178375787	2.33E-18
Feeling abnormal	6.12E-10	1.74E-06	6.12E-10
Rash	0.0005803	6.39E-86	0.000580257
Condition aggravated	5.93E-22	2.09E-29	5.93E-22
Chest discomfort	8.24E-07	0.59509708	8.24E-07
Arthralgia	6.41E-05	2.39E-34	6.41E-05
Paraesthesia	0.5446063	2.02E-46	0.544606299
Unresponsive to stimuli	1.63E-12	0.019741655	1.63E-12
Diarrhoea	9.25E-07	9.38E-43	9.25E-07
Pruritus	1.66E-05	2.70E-92	1.66E-05
Heart rate increased	0.3560273	7.15E-17	0.356027306
Urticaria	1.65E-05	1.59E-80	1.65E-05
Facial paralysis	0.0008205	7.78E-09	0.000820484
Syncope	0.0426228	2.57E-10	0.042622813
Tachycardia	1.09E-13	4.25E-13	1.09E-13
Palpitations	0.0078099	4.50E-21	0.007809903
Hyperhidrosis	0.5857873	1.16E-12	0.585787253
Erythema	4.86E-06	1.10E-78	4.86E-06
Throat tightness	0.0066261	8.57E-12	0.006626105
Tremor	0.733015	1.60E-10	0.733015003
Blood pressure increased	2.90E-06	5.31E-07	2.90E-06
Anaphylactic reaction	0.0001342	6.95E-10	0.000134246
Intensive care	2.05E-75	4.19E-126	2.05E-75
Loss of consciousness	0.044628	5.26E-12	0.044628039
Decreased appetite	1.70E-07	5.11E-23	1.70E-07

Muscular weakness	1.88E-09	0.000401598	1.88E-09
Flushing	0.8975687	2.58E-12	0.89756871
Mobility decreased	0.0551621	1.35E-13	0.055162127
Injection site erythema	0.9784857	5.58E-31	0.978485699
Feeling hot	0.0650965	3.32E-15	0.065096524
Abdominal pain	3.39E-13	1.12E-09	3.39E-13
Injection site swelling	0.5166127	2.20E-31	0.516612685
Cerebrovascular accident	8.09E-37	0.539062628	8.09E-37
Cardiac arrest	4.96E-05	1.05E-12	4.96E-05
Lymphadenopathy	0.0005726	6.70E-35	0.000572649
